# Editorial: New Insights Into the Complexity of Tumor Immunology in B-Cell Malignancies: Disease Biology and Signaling

**DOI:** 10.3389/fonc.2021.820984

**Published:** 2021-12-15

**Authors:** Jérôme Paggetti, Martina Seiffert, Etienne Moussay

**Affiliations:** ^1^ Tumor-Stroma Interactions, Department of Cancer Research, Luxembourg Institute of Health, Luxembourg, Luxembourg; ^2^ Molecular Genetics, German Cancer Research Center (DKFZ), Heidelberg, Germany

**Keywords:** leukemia, lymphoma, tumor microenvironment, stromal cells, CLL (chronic lymphocytic leukemia), immunotherapy, anti-tumor immunity

Over the last decade, the interactions between neoplastic lymphocytes and the tumor microenvironment, especially the immune system and stromal cells, have proven to be key in the pathogenesis of B-cell neoplasms, including chronic lymphocytic leukemia (CLL), acute lymphoblastic leukemia, Hodgkin and Non-Hodgkin lymphomas (NHL), and multiple myeloma. In these malignancies, tumor cells in the bone marrow, lymph nodes, spleen or in peripheral blood are by nature surrounded by immune cells such as B and T lymphocytes, regulatory T cells, monocytes, macrophages, and neutrophils, which were shown to harbor tumor-supportive and immunosuppressive features. Hence, understanding the complex crosstalk between malignant and non-malignant cells in lymphoid malignancies is crucial to design innovative therapeutic strategies, including immunotherapy. To this end, an in-depth characterization of tumor-associated immune cells and other accessory cells and their complex interactions with malignant cells within the tumor microenvironment will be crucial to identify new therapeutic targets and to develop successful combination treatment strategies including immunomodulatory drugs. In this first volume, 13 articles explore disease biology and signaling in different B-cell malignancies and their connection to the microenvironment.

CLL cells are mainly quiescent in the peripheral blood but a small proliferative pool exists in the lymph nodes. In a timely review (1), Haselager, Kater and Eldering explain the different signals implicated in CLL proliferation and their connections to the lymph node microenvironment. They stress on the importance of the B cell receptor (BCR) activation in CLL cell growth and the therapeutic targeting of this pathway using inhibitors of the Bruton’s tyrosine kinase (BTK). However, *in vitro* BCR triggering is unable to generate significant cell proliferation, highlighting the complexity of the *in vivo* situation and the need for better *in vitro* modelling to mimic the disease. This is the main focus of a mini review (2) by Scielzo and Ghia in which they highlight the current lack of a suitable culture model in B-cell malignancies compared to solid tumors. They detailed the current innovation regarding static and dynamic 3D culture in which accessory cells can be introduced with the goal to better reflect the *in vivo* microenvironment. These models should be further developed and will become indispensable to evaluate the potency of novel drugs *in vitro*.

As stated above, BCR activation is a key component of B-cell malignancies. In a dedicated review (3), Thurner, Bewarder and colleagues discuss the current understanding of specific antigens recognized by the BCR of various lymphomas. These antigens can be exogenous from bacteria or autoantigens which are mostly the results of abnormal post-translational modifications. This knowledge could lead to the development of new therapies exploiting the recognition of lymphoma cells *via* these specific peptides.

CLL cells are highly dependent on their interactions with the microenvironment. This is illustrated by the fact that primary CLL cells undergo rapid and spontaneous apoptosis when cultured *in vitro*, but survival is promoted when stromal cells are added to the culture. In a very detailed review (4), Dubois, Stamatopoulos and colleagues explained the bidirectional crosstalk between stromal cells, mainly mesenchymal stem cells and follicular dendritic cells, and malignant cells which support leukemic cell survival, chemotaxis and homing through the activation of specific signaling pathways. Hence, targeted therapies disrupting this multifaceted crosstalk could abolish the stroma-supportive effects and favor elimination of the leukemic clone. Myeloid cells are also important players in the microenvironment of B-cell malignancies. In an original research article (5), Farnworth-McHugh, Gregory and colleagues demonstrated that in starry-sky B-cell lymphoma, tumor-associated macrophages accumulate and display phagocytic activity towards apoptotic lymphoma cells supporting their pro-oncogenic phenotype. The receptor tyrosine kinase MERTK is critical in this phenomenon, opening novel therapeutic opportunities with targeted kinase inhibitor.

Understanding the complex relationship between different lymphoma entities and their ecosystem, especially in the light of tumor heterogeneity, is fundamental to unravel specific vulnerabilities and design efficient (immuno) therapies. Ysebaert, Fournié and colleagues explain in an informative review (6), how single-cell transcriptomic technologies can be used to tackle these problematics. They also discuss how these novel technical approaches can be used in clinical trial to monitor treatment response and the development of resistance and toxicity with the aim to foster precision medicine.

Metabolism rewiring is an important feature of cancer development. Böttcher, Mougiakakos and colleagues described in a comprehensive mini review (7), the metabolic alterations found in CLL and different B-cell NHL, and how they can impact anti-lymphoma immunity. Indeed, they introduced a rather novel concept of immunometabolic regulation where lymphoma and immune cells communicate through metabolic reprogramming, creating novel dependencies that could be therapeutically exploited. In the future, efforts should be undertaken to decipher these metabolic pathways with the aim to target lymphoma cell vulnerabilities and at the same time reactivate immune cells with a more favorable microenvironment.

In a complementary review (8), Domka, Goral and Firczuk addressed the dual role of reactive oxygen species (ROS) in B-cell malignancies. Malignant B cells display an imbalanced redox homeostasis that require their metabolic adaptation which is in part supported by stromal cells. ROS have also detrimental effects on immune cells affecting the anti-tumor immune response. Therapies targeting the antioxidant system could therefore represent novel approaches in B-cell malignancies.

ZAP-70 is an important tyrosine kinase mainly implicated in T-cell receptor activation which can be aberrantly expressed in different B-cell malignancies, in particular in a subset of CLL patients associated with unfavorable outcome. In a focused review (9), Chen, Moore and Ringshausen explored the recent evidence demonstrating that beyond its intrinsic role in malignant B cells, ZAP-70 can also influence the crosstalk between tumor cells and their microenvironment, especially the immune cells. They also reviewed the function of ZAP-70 in T cells’ and NK cells’ anti-tumor immunity emphasizing the need of future studies to evaluate the benefit or harm of ZAP-70 therapeutic targeting in the frame of B-cell malignancies.

Another crucial enzyme in B cells is AID (activation-induced cytidine deaminase) which is implicated in immunoglobulin gene diversification. In a focused review (10), Oppezzo, Navarrete and Chiorrazi explained the detrimental role of AID in leukemogenesis and disease progression by its “off-target” mutagenic activity, and also how AID is regulated in the lymph node microenvironment. In a mini review (11), Munguia-Fuentes, Maqueda-Alfaro, Yam-Puc and colleagues described the microenvironment of lymphoid tissue germinal centers where B cells undergo antigen-driven somatic hypermutations, a mechanism that is associated with malignant transformation.

Sustained NF-κB activation is key in the pathogenesis of B-cell lymphomas. The adaptor protein MYD88 is an important player in the Toll-Like receptors signaling pathway which can be activated by the microenvironment and leads to NF-κB activation. In an original research article (12), Cardona-Gloria, Weber and colleagues identified different splice variants of MYD88 with variable signaling capacities in human B cells. Importantly, they demonstrated that malignant B cells favor splice variants that have NF-κB promoting activities. Contrary to myeloid cells, the negative feedback loop leading to the synthesis of variants with no signaling activities is absent in B cells, potentially explaining their susceptibility to lymphoma transformation.

Telomeres maintenance is also crucial for tumorigenesis. In a detailed review (13), Jebaraj and Stilgenbauer summarized the current knowledge on telomere length, telomerase activity, and associated proteins of the shelterin complex in CLL. All these components are dysregulated in CLL with a complex interconnection underlying their role in different phases of disease development. Nevertheless, studies should be conducted to evaluate the intrinsic and extrinsic signals leading to these modulations and the associated mechanisms in order to therapeutically target them.

With this Research Topic dealing with the microenvironment of B-cell malignancies, in particular its connection to disease biology along with the signaling pathways involved, we hope to bring to the readers a timely and interesting overview of how the microenvironment shape the development and progression of these diseases independently of the genetic mutations of the tumor clone.

## Author Contributions

All authors edited the Research Topic. JP wrote the editorial. All authors contributed to the article and approved the submitted version.

## Funding

This work was supported by grants from Fonds National de la Recherche Luxembourg to JP and EM (C20/BM/14592342 and C20/BM/14582635).

## Conflict of Interest

The authors declare that the research was conducted in the absence of any commercial or financial relationships that could be construed as a potential conflict of interest.

## Publisher’s Note

All claims expressed in this article are solely those of the authors and do not necessarily represent those of their affiliated organizations, or those of the publisher, the editors and the reviewers. Any product that may be evaluated in this article, or claim that may be made by its manufacturer, is not guaranteed or endorsed by the publisher.
